# Detecting disease-causing genes by LASSO-Patternsearch algorithm

**DOI:** 10.1186/1753-6561-1-s1-s60

**Published:** 2007-12-18

**Authors:** Weiliang Shi, Kristine E Lee, Grace Wahba

**Affiliations:** 1 Department of Statistics, University of Wisconsin Madison, 1300 University Avenue, Madison, Wisconsin 53706, USA; 2 Department of Ophthalmology & Visual Sciences, University of Wisconsin Madison, 610 North Walnut, 4th Floor WARF, Madison, Wisconsin 53726, USA; 3 Departments of Statistics, Biostatistics and Medical Informatics and Computer Sciences, University of Wisconsin Madison, 1300 University Avenue, Madison, Wisconsin 53706, USA

## Abstract

The Genetic Analysis Workshop 15 Problem 3 simulated rheumatoid arthritis data set provided 100 replicates of simulated single-nucleotide polymorphism (SNP) and covariate data sets for 1500 families with an affected sib pair and 2000 controls, modeled after real rheumatoid arthritis data. The data generation model included nine unobserved trait loci, most of which have one or more of the generated SNPs associated with them. These data sets provide an ideal experimental test bed for evaluating new and old algorithms for selecting SNPs and covariates that can separate cases from controls, because the cases and controls are known as well as the identities of the trait loci. LASSO-Patternsearch is a new multi-step algorithm with a LASSO-type penalized likelihood method at its core specifically designed to detect and model interactions between important predictor variables. In this article the original LASSO-Patternsearch algorithm is modified to handle the large number of SNPs plus covariates. We start with a screen step within the framework of parametric logistic regression. The patterns that survived the screen step were further selected by a penalized logistic regression with the LASSO penalty. And finally, a parametric logistic regression model were built on the patterns that survived the LASSO step. In our analysis of Genetic Analysis Workshop 15 Problem 3 data we have identified most of the associated SNPs and relevant covariates. Upon using the model as a classifier, very competitive error rates were obtained.

## Background

Rheumatoid arthritis (RA) is a complex disease with a moderately strong genetic component. Generally, females are at a higher risk than males and the mean onset of disease is in the fifth decade. Many studies have implicated the HLA region on 6p21 with consistent evidence for several of the DR alleles contributing to risk. There remains much to learn about the genetic susceptibility for rheumatoid arthritis and possible gene and environmental interactions.

Identification of disease-causing genes requires extensive evaluation of multiple potential genetic sites. The current trends in genetic epidemiology are to evaluate thousands of single-nucleotide polymorphisms (SNPs) along the chromosome to identify regions where the true disease-causing gene may lie. Tree-structured methods such as CART (classification and regression trees) [1] and Logic regression [2] usually select variables sequentially, and hence may miss the overall correlation structure of the variables. Random forest [3], which grows a large number of classification or regression trees with no trimming or pruning, has gained popularity in the analysis of genetic data. More recently a forward-stepwise penalized logistic regression [4] has been developed for screening gene-gene interactions, which is also a sequential method. We will be using the penalized likelihood method with the LASSO penalty to select SNPs, gene × gene interactions, and gene × environmental interactions. For Gaussian data the LASSO [5] was proposed as a variant of linear least-squares ridge regression. It was demonstrated that this approach tended to set many of the coefficients to zero, resulting in a sparse model, a property not generally obtained with quadratic penalties. LASSO-Patternsearch algorithm [6] was proposed to search patterns of multiple risk factors in large demographic studies. The core of the method is global, in that it deals with a very large number of patterns simultaneously, as opposed to sequential methods that constitute much of the literature in this area. In this paper, we applied the modified LASSO-Patternsearch algorithm on the simulated RA data from Genetic Analysis Workshop 15 (GAW15). The method has been modified in three places. First, we introduce a screen step to eliminate most of the noise SNPs and their interactions before applying the LASSO step. Second, we only consider the main effects and second-order interactions for computation and interpretation. And last, we take advantage of the fact that we can extract separate training, tuning, and test data sets, all generated from the same (simulated) population. Therefore, we choose the tuning parameters by prediction accuracy on the tuning set, and, for quantitative comparison with other methods, estimate the prediction accuracy of the resulting model on the test set.

## Methods

### Data set

We have chosen to use the simulated data (Problem 3) from GAW15. This data simulation was set up to mimic the familial pattern of RA, including a strong effect of DR type at the HLA 2 locus on chromosome 6. A large population of nuclear families (two parents and two offspring) was generated. This population contains close to 2 million sibling pairs (3.6 million subjects). RA affection status was determined for everyone from a complex genetic and environmental model. There were four loci (A on chromosome 16, B on chromosome 8, and C and D both on chromosome 6) in addition to a strong effect of the DR alleles that directly, or through interactions with smoking and gender, modeled RA status. Additional loci modeled severity and other related RA outcomes. From this population, a random sample of 1500 families was selected from among families with two affected offspring (the affected sib-pair (ASP) group) and another random sample of 2000 families was selected from among families where no member was affected (control group). Within the 2000 families selected for the control group, one offspring was randomly selected to be in the final control group.

Microsatellites and SNPs were generated on 22 autosomes. These markers were designed to be like real human autosomes in terms of genetic and physical map lengths. The marker and trait loci were generated to have similar properties, such as linkage disequilibrium, to those observed in real data. We chose to analyze the SNPs, for all controls and the first sibling in the ASP group in Replicate 1. In addition, we used similar data from Replicates 2 and 3 as tuning and test data sets.

### LASSO-Patternsearch algorithm

The LASSO-Patternsearch algorithm [6] is an approach to identify patterns of risk factors that is built on a global core. We modify the original algorithm for use with genetic (SNP) data (add a screen step, consider only main effects and second-order interactions, and tune the smoothing parameters with a tuning set). Through the use of a series of basis functions described below, we can build a model for the relation between phenotype and variables that embodies main effects and two-factor interactions ("patterns"). The basis functions we use assume dichotomous risk factors. Responses are coded 1 for cases and 0 for controls; females are coded 1 and males 0; smokers as 1 and non-smokers as 0. Age is the only continuous risk factor and we code an elder group (≥ 55) as 1 and a younger group (<55) as 0. Because nearly all SNPs have three levels: normal, one variant allele, and two variant alleles, we retain this information by initially coding them as 0, 1, and 2, respectively. HLA DR also has three levels and we initially code them as DRX = 0, DR1 = 1, and DR4 = 2. For these three level variables, we define basis functions in a generalized way described below, which is equivalent to introducing two dummy variables.

The modified algorithm has three steps:

• Step 0: The Screen step

We first define our coding basis functions to be used: let *x*_*j *_be the *j*^th ^variable and *x *be (*x*_1_, *x*_2_,..., *x*_*N*_) where *N *is the number of variables. Let $B_{j}^{1}(x)=1$ if *x*_*j *_= 1, and 0 otherwise, and let $B_{j}^{2}(x)=1$ if *x*_*j *_= 2, and 0 otherwise. We call these "main effects" basis functions. Let $B_{jk}^{rs}(x)=1$ if *x*_*j *_= *r*, *x*_*k *_= *s*, *r*, *s *= 1, 2, and 0 otherwise (so there are four basis functions for each pair (*j*, *k*)). We call these "two-factor interaction" basis functions. These basis functions will be used to code the variables into logistic or penalized logistic regression models. Let *p*(*x*) be the probability that *y *= 1, given *x*, and let *f*(*x*) = log[*p*(*x*)/(1 - *p*(*x*))]. The negative log likelihood function is given by:

$$L(y,f)=\sum_{i=1}^{n}\left[-y_{i}f(x(i))+\log(1+e^{f(x(i))})\right].$$

We will code the dependence on *x *by *f*(*x*) = *μ *+ ∑*c*_ℓ_*B*_ℓ_(*x*), where the *B*_ℓ _will be specified subsets of the basis functions defined above, and *μ *and {*c*_ℓ_} are estimated by minimizing *L*(*y*, *f*). The goal is to select those basis functions that encode the variables or pairs of variables that best separate cases from controls.

Because there are more than 9000 SNPs on all 22 chromosomes, incorporating 9000 main effects and $\left(\begin{array}{c} 9000\\ 2 \end{array}\right)$ two-factor interactions, there will be more than 10^8 ^basis functions and we cannot deal with them all simultaneously. We first prescreen for main effects with a logistic regression model as follows. For each *j *= 1,..., *N*, we find *μ*, $c_{j}^{1}$ and $c_{j}^{2}$ to minimize the negative log likelihood *L*(*y*, *f*_*j*_), where $f_{j}(x)=\mu_{j}+\sum_{r=1}^{2}c_{j}^{r}B_{j}^{r}(x)$. We test the hypothesis at the 0.05 level that $c_{j}^{r}$ is different from 0 and if it is, the *j*^th ^variable will go to the second part of the prescreen step and the basis function $B_{j}^{r}$(*x*) will go to Step 1. Note that each SNP may contribute two basis functions and they are not necessarily significant simultaneously. In that case, the significant basis function will go to the LASSO step and the SNP is still eligible for the screening of interactions. For each pair of variables (*x*_*j*_, *x*_*k*_), we construct the model $f_{jk}(x)=\mu_{jk}+\sum_{r=1}^{2}c_{j}^{r}B_{j}^{r}(x)+\sum_{s=1}^{2}c_{j}^{s}B_{k}^{s}(x)+\sum_{r,s=1,s}c_{kj}^{rs}B_{jk}^{rs}(x)$ and minimize *L*(*y*, *f*_*jk*_). We test the hypotheses that the coefficients $c_{jk}^{rs}r,s=1,2$ are different from 0 at the 0.002 level and the basis functions (patterns) that survive go to Step 1. At this point we would like to select the largest number of candidates that can be comfortably handled by the core global LASSO step. The significance level of 0.002 was chosen in an *ad hoc *manner to select candidates for the next step and resulted in a large set that, very roughly, met this goal.

• Step 1: The LASSO step

In this step, we use the LASSO penalty (*l*_1_) to do variable selection. We relabel the basis functions that survive Step 0 as *B*_*l*_, *l *= 1, 2..., *N*_*B*_, where *N*_*B *_is the total number of the basis functions. We estimate *f *by minimizing

*I*_*λ *_(*y*, *f*) = *L*(*y*, *f*) + *λJ*(*f*),

where $f(x)=\mu +\sum_{\ell =1}^{N_{B}}c_{\ell }B_{\ell }(x)$ and $J(f)=\sum_{\ell =1}^{N_{B}}\left|c_{\ell }\right|$. The smoothing parameter *λ *balances the trade-off between data fitting and the sparsity of the model. We will choose the smoothing parameter by the prediction accuracy on a separate tuning set. This is done as follows: for each trial value of *λ*, the minimizer of Eq. (2) produces *f*_*λ *_(*x*) and hence *p*_*λ *_(*x*). We make the important observation that the ratio of cases and controls in the training set is *the same *as the ratio of cases and controls in the tuning set. Thus, if one had a perfect estimate of *p*(*x*) *for the population that generated both the test and tuning set*, and costs of misclassification were the same for both types of misclassification, then the Bayes rule (to minimize expected cost) for classifying members of the tuning set would be to classify a member as case if *p*(*x*) > 0.5 and as a control if *p*(*x*) < 0.5 [7]. Therefore we are motivated to examine the actual error rates on the tuning set, for each choice of *λ*, by using *p*_*λ*_(*x*) = 0.5 as the classifier.

• Step 2: The logistic regression step

Step 1 produces a relatively sparse model, but we have seen a general tendency for the LASSO-Patternsearch to err on the conservative side in selecting basis functions, that is, there is a very high probability of including all relevant basis functions, at the expense of including some noise terms [6,8]. Thus, we took a closer look at the terms that passed Step 1 by putting them into a parametric logistic regression and testing the significance of each term at level *α*. Rather than choose *α *on an ad hoc basis, it is selected based on prediction accuracy on the tuning set. The significant term goes into the logistic regression model again and that gives the final model.

It is believed that this multi-step process is an effective procedure to meet two goals simultaneously, sparsity and generalizability, and the results below tend to bear this out.

## Results

We selected the first replicate as the training set, the second replicate as the tuning set, and the third replicate as the test set. In our first pass, we examined age, smoking, and sex as environmental factors, and all chromosome 6 SNPs. The screen step identified 145 main effects and 1439 interactions, while the final model included only 6 main effects and no interactions (Table 1). We found SNP6_153–SNP6_154, which we later (after obtaining the answers) found out were close to locus C, and SNP6_162, which was close to locus D. We also found sex and smoking as expected. Applying this model to predict the RA cases in Replicate 3 as any with an estimated probability >0.5 resulted in a 13.8% error rate, with sensitivity of 85.3% and specificity of 87.0%. In fact, a plot of the prediction error rate as a function of the threshold (not shown) is essentially a convex curve with a minimum of 13.8% for any *p *between 0.41 and 0.56, verifying the appropriateness of the use of *p *= 0.5 as the threshold.

**Table 1:** Model on chromosome 6

Variable	No. variant alleles at locus	Coefficient^a^	SD	*p*-Value
Smoking	-	0.8653	0.1088	10^-15^
Sex	-	1.0478	0.1131	10^-20^
SNP6_153	1	-2.0411	0.1365	10^-50^
SNP6_154	1	-1.4509	0.1448	10^-23^
SNP6_162	1	2.2297	0.2767	10^-16^
SNP6_153	2	-5.5977	0.2707	10^-95^

^a^Coefficients are estimated in the final parametric logistic regression model.

We then expanded our analysis to SNPs on all chromosomes and included the DR allele from each parent. That gave us 9192 variables, including 9187 SNPs, two DR alleles from parents, age, smoking, and sex. The main effect screen in Step 0 identified 880 basis functions, corresponding to 795 variables. We then screened for the interaction of these 795 variables and got 1679 interactions. Step 1 included 2559 (880 + 1679) basis functions. The final model identified eight main effects and three interactions (listed in Table 2 and 3). The main effects include DR allele from the parents, gender, and smoking, as well as the SNPs from chromosome 6 and an additional SNP on chromosomes 11. All of these were modeled in the simulation: SNP6_154 is close to locus C, SNP6_162 is close to locus D, and SNP11_389 is close to locus F (which modeled severity of IgM). We have also identified three interaction terms, including one within-chromosome interaction on chromosome 2 and two between-chromosome interactions. These interactions were not directly modeled in the simulation. The prediction error of this model on Replicate 3 is 12.6%, with sensitivity of 85.5% and specificity of 88.8%. A plot of the prediction error as a function of the threshold is a convex curve with the minimum error rate of 12.6% for any *p *between 0.49 and 0.51.

**Table 2:** Main effects model on all chromosomes

Variable	Level	Coefficient	SD	*p*-Value
Smoking	-	1.0434	0.1214	10^-18^
Sex	-	1.0819	0.1251	10^-18^
SNP6_154	1	-1.6228	0.1395	10^-31^
SNP6_162	1	2.2717	0.2885	10^-15^
HLA DR type, father	2	2.3848	0.1405	10^-64^
HLA DR type, mother	2	2.3443	0.1388	10^-64^
SNP6_154	2	-3.0081	0.5492	10^-8^
SNP11_389	2	0.9521	0.1264	10^-14^

^a^For SNPs, level is the number of variant alleles.  For DR type, level = 1 means DR1 and level = 2 means DR4.

**Table 3:** Interactions on all chromosomes

Variable 1	No. variant alleles of Variable 1	Variable 2	No. variant alleles of Variable 2	Coefficient	SD	*p*-Value
SNP2_542	1	SNP2_768	1	-0.5061	0.1389	0.0003
SNP1_673	1	SNP15_77	1	-0.8369	0.1693	10^-7^
SNP8_233	1	SNP16_131	2	-0.8044	0.1633	10^-7^

Our method successfully selected many trait loci, but it also missed some. Locus B is on chromosome 8 and it increases the RA risk for smokers. We did not find this because locus B is at the end of the chromosome and none of the SNPs are close by. We also missed locus A, which affects the impact of HLA DR types. Another interaction we missed is sex and locus C. We tabulate the raw data in Table 4. According to the solution and the relationship between locus C and SNP6_154, we should see no difference between males and females when SNP6_154 = 2. Females will be at higher risk than males when SNP6_154 = 1 and the difference is even bigger when SNP6_154 = 0. However, there are very few cases when SNP6_154 = 2. We cannot really tell whether there is a difference at this level. Plus, locus C has a strong correlation with the DR allele. Therefore, we ended up with the main effects of sex and SNP6_154 rather than their interaction.

**Table 4:** The raw data of sex and SNP6 15. The denominator is the total number of people and the numerator is the total number of RA patients.

SNP6154	Male	Female
0	341/551 = 0.619	1015/1241 = 0.818
1	43/520 = 0.083	97/628 = 0.154
2	1/270 = 0.004	3/290 = 0.010

## Discussion

The LASSO-Patternsearch algorithm [6] was originally designed for demographic studies in which the data sets are smaller with fewer variables. It is a two-stage method whose core is global, as opposed to sequential methods, like trees and forward-stepwise penalized logistic regression [4]. We added a screen step to the front end here to handle the extremely large number of potential SNP patterns. We roughly maximized the number of patterns surviving this step within the limits of the core LASSO step, which can handle 4000 basis functions. We believe that this conservative screen step is unlikely to delete important patterns here. Proof would await our ability to handle larger numbers of basis functions but the results in selecting relevant SNP patterns here certainly support that belief. The LASSO step took in the resulting large number of basis functions and returned a small fraction of them, retaining the flavor of a completely global algorithm, with the final tuning step removing less significant patterns, chosen as to maximize classification accuracy on the tuning set. The LASSO-Patternsearch method is complementary to random forest approaches. The random forest method is global, but operates quite differently. Thus, LASSO-Patternsearch provides a complimentary tool for the data analyst dealing with very large attribute vectors. LASSO-Patternsearch is also very efficient. Run time for the LASSO step with 2559 basis functions was 30 minutes on our system (3.4 GHz CPU, 3.7 GB memory). Speed and capacity of the algorithm compare well with other methods discussed. Our method was able to identify important SNPs and covariates, and separate cases from controls similar to the best results presented at the meeting. We believe that it provides a useful new tool for the analysis of genetic data.

## Competing interests

The author(s) declare that they have no competing interests.
